# Combination of Superselective Arterial Embolization and Radiofrequency Ablation for the Treatment of a Giant Renal Angiomyolipoma Complicated with Caval Thrombus

**DOI:** 10.1155/2016/8087232

**Published:** 2016-05-11

**Authors:** Konstantinos N. Stamatiou, Hippocrates Moschouris, Kiriaki Marmaridou, Michail Kiltenis, Konstantinos Kladis-Kalentzis, Katerina Malagari

**Affiliations:** ^1^Department of Urology, Tzaneio General Hospital, 18536 Piraeus, Greece; ^2^Department of Diagnostic and Interventional Radiology, Tzaneio General Hospital, 18536 Piraeus, Greece; ^3^2nd Department of Radiology, University of Athens, Attikon Hospital, Chaidari, 12462 Athens, Greece

## Abstract

This is a case of a 78-year-old male patient with multiple angiomyolipomas of a solitary right kidney. The largest of these tumors (maximum diameter: 13.4 cm) caused significant extrinsic compression of the inferior vena cava complicated by thrombosis of this vessel. Treatment of thrombosis with anticoagulants had been ineffective and the patient had experienced a bleeding episode from the largest right renal angiomyolipoma, which had been treated by transarterial embolization in another institution, 4 months prior to our intervention. Our approach included superselective transarterial embolization of the dominant, right kidney angiomyolipoma with hydrogel microspheres, which was combined, 20 days later, with ultrasonographically guided radiofrequency ablation. Both interventions were uneventful. Computed tomography 2 months after ablation showed a 53% reduction in tumor volume, reduced space-occupying effect on inferior vena cava, and resolution of caval thrombus. Nine months after intervention the patient has had no recurrence of thrombosis or hemorrhage and no tumor regrowth has been observed. The combination of superselective transarterial embolization and radiofrequency ablation seems to be a feasible, safe, and efficient treatment of large renal angiomyolipomas.

## 1. Introduction

Angiomyolipomas (AMLs) have been classified among the perivascular epithelioid cells tumor group (PEComas). They are composed of variable amounts of three components: blood vessels (angioid), smooth muscle (myoid), and mature fat (lipoid) components. AMLs represent the most common benign, noncystic renal lesion [1]. Two types have been described: sporadic and multiple. The first occurs as a single tumor in one kidney. It accounts for 80% of renal AMLs and it is typically identified in adults, with a strong female predilection. The second occurs as larger tumor and/or multiple tumors in both kidneys and accounts for 20% of renal AMLs. It affects both sexes at a younger age than sporadic AML. It is seen in association with tuberous sclerosis and lymphangioleiomyomatosis. AMLs are benign and usually asymptomatic. Although most AMLs are incidentally diagnosed on cross-sectional imaging, they do have the risk of rupture with bleeding or secondary damage of surrounding structures, as they grow. The risk of bleeding and surrounding tissue damage is proportional to the size of the lesion (diameter > 4 centimeters). AMLs may also be associated with palpable mass, flank pain, urinary tract infections, haematuria, renal failure, hypertension, and, rarely, renal vein and/or inferior vena cava thrombosis. AMLs found incidentally are usually small and so require no therapy. Lesions that present with retroperitoneal hemorrhage often require emergency transarterial embolization as a life-saving measure [2]. Although embolization is effective for this purpose, some authors report a significant percentage of recurrent hemorrhage, recurrent symptoms, or inadequate tumor shrinkage after embolization [3].

We herein describe a complicated case of a giant renal AML, which was successfully managed by a combined interventional radiologic approach.

## 2. Case Presentation

**Figure 1:**
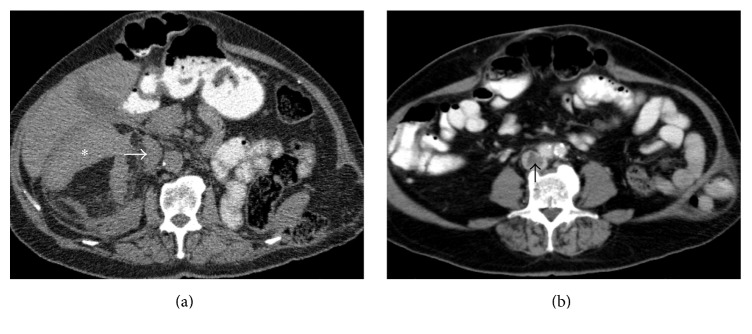
Axial CT images prior to intervention. (a) Unenhanced image shows the typical appearance of a large angiomyolipoma with fat and soft-tissue (*∗*) densities. The mass compresses the inferior vena cava (arrow). (b) Contrast-enhanced image (venous phase) shows a thrombus causing an enhancement defect (arrow) at the lowest part of the inferior vena cava.

A 78-year-old male patient presented to our institution with a history of complicated renal AMLs. One year earlier, the patient had undergone a left nephrectomy for the management of massive spontaneous hemorrhage from a giant AML of the ipsilateral kidney. The right kidney was affected by several small (<2 cm) AMLs and by a dominant tumor measuring 13.4 × 10.5 × 7.5 cm. The composition of this tumor was primarily fat, with a soft-tissue component at the upper pole of the tumor. This component caused significant extrinsic compression of the inferior vena cava, and a thrombus was observed at the lower part of this vein (Figure 1). Treatment of the caval thrombus with anticoagulants (acenocoumarol per os, with target International Normalized Ratio of 2.5) for 5 months had proven ineffective. During that period, the patient had experienced an episode of severe hemorrhage from the dominant tumor of the right kidney, which was managed with transarterial embolization in another institution. The patient also complained of moderate right flank pain. The rest of his medical history was unremarkable and there were no clinical or imaging findings indicative of a congenital disorder.

**Figure 2:**
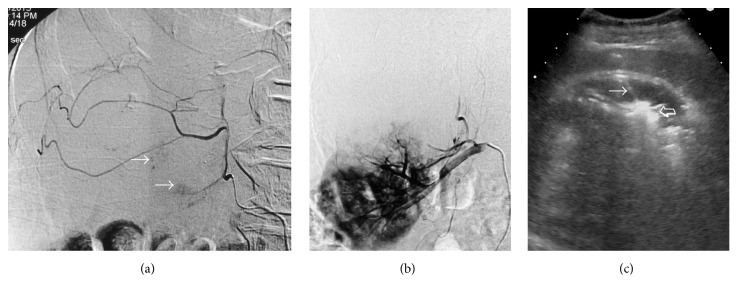
Representative images from the interventions. (a) Digital subtraction angiography (DSA) image after superselective catheterization of the tumor feeders shows relatively few and thin tumoral arteries and a limited tumor blush (arrows). (b) DSA image after embolization shows devascularization of the tumor with preservation of the renal enhancement. (c) Coronal oblique sonographic image during ablation shows the electrode (arrow), which has been advanced into the soft-tissue part of the tumor. Strong echoes (open arrow) caused by tissue vaporization are noted around the electrode tip.

The patient was evaluated by a multidisciplinary team consisting of urologist, interventional radiologist, nephrologist, vascular surgeon, and pathologist. It was decided to utilize a combined interventional treatment, in order to reduce the risk of future hemorrhage, achieve a degree of tumor shrinkage, and minimize the adverse effects on the integrity and function of the solitary right kidney. Superselective arterial embolization (SAE) was performed first. Vascular access was gained via the right common femoral artery, with Seldinger technique, and, after a flush aortogram, the right renal artery was selectively catheterized with a 5-French, Cobra-1 angiographic catheter. The large upper pole AML was fed by a few, relatively thin arteries arising from the right renal capsular artery. No aneurysms or arteriovenous shunts were observed. Superselective approach was achieved by means of 2.2-French Microcatheter (Stridesmooth, Asahi Intecc Co. Ltd., Aichi, Japan) and embolization was performed with tightly calibrated, hydrogel microspheres (Embozene, Celonova BioSciences, San Antonio, Texas, USA) with diameters of 250 and 400 micrometers (*μ*m). Postembolization angiogram showed devascularization of the AML and no signs of renal infarction. With the exception of right flank pain, the procedure was well tolerated and the patient was discharged the following day. A radiofrequency ablation (RFA) of the dominant angiomyolipoma of the left kidney was performed 20 days later (Figure 2). With the patient in left lateral decubitus position, an oblique coronal sonogram of the tumor was acquired and the upper portion of the tumor (which compressed the IVC) was targeted. A 17-gauge, water cooled, radiofrequency electrode with a 3 cm active tip was utilized (Jet-Tip, RF Medical Co., Seoul, Korea). The electrode was successively inserted at two sites at the upper portion of the tumor and a 12-minute ablation cycle was applied to each site. The RF power output was automatically adjusted by the generator and ranged from 60 to 140 Watts. During ablation, a slow infusion of 20 mL of normal saline was performed by means of a dedicated pump, through the electrode into the tumoral tissue, to improve coagulation and to prevent carbonization. Medication for pain control included ropivacaine hydrochloride (15 mL of Naropeine 0.2%, as local injection) and pethidine (100 mg/2 mL, as slow intravenous infusion). The ablation was monitored ultrasonographically. At the end of the procedure, scanning at multiple planes showed that the largest part of the upper pole of the tumor had been covered by high-level echoes (ablation-related gas formation). Finally, the electrode tract was ablated to decrease the risk of bleeding. The patient received intravenous hydration and antibiotics and was discharged the following day.

**Figure 3:**
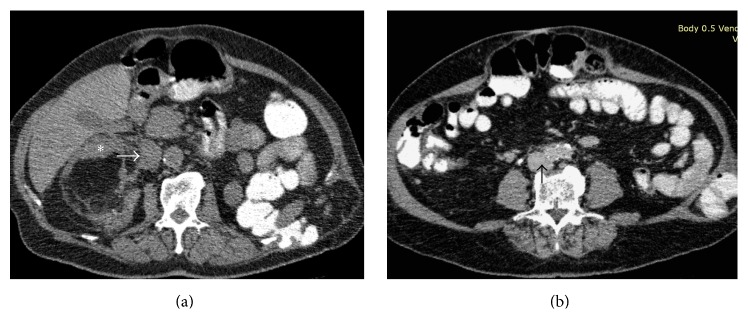
Axial CT images after embolization and ablation. (a) Unenhanced image shows that the angiomyolipoma is smaller, with relative shrinkage of the soft-tissue component (*∗*) in favor of the fat. The compression of the IVC (arrow) is now less striking. (b) Contrast-enhanced image (venous phase) shows disappearance of the caval thrombus (arrow).

A computed tomography (CT) scan performed 8 weeks after RFA revealed shrinkage of the dominant AML of the right kidney, with 17% reduction in maximum tumor diameter and 53% reduction in tumor volume. There was a significant (75%) reduction in the volume of the soft-tissue component. The extrinsic compression of the IVC was also relieved and complete resolution of the caval thrombus was noted (Figure 3). Three months after intervention, acenocoumarol treatment was terminated. Nine months posttreatment the patient reported partial resolution of the right flank pain and sonography showed a minimal further decrease in maximum tumor diameter and no evidence of IVC thrombus. Serum creatinine values were near the upper normal limits prior to treatment and throughout the follow-up period.

## 3. Discussion

To manage both the hemorrhagic potential and the space-occupying effect of the large AML of our case, we adopted a combined interventional radiologic approach.

SAE was performed first, in order to devascularize the tumor and to reduce the risk of hemorrhage (either spontaneous or iatrogenic). SAE is a widely accepted intervention for the treatment of symptomatic AMLs and for prophylactic treatment of asymptomatic AMLs larger than 4 cm [2]. A variety of embolic agents have been used for SAE of AML and no widely accepted guidelines exist. In our institution we treat non-aneurysm bearing AMLs exclusively with the aforementioned type of microspheres. The properties of these microspheres (spherical shape, smooth surface, tight diameter calibration, and insignificant inflammatory reaction of surrounding tissues) allow for predictable, complete, and well tolerated vessel occlusion. Regarding the selected size of the microspheres, we, like others [2], have concerns about the safety of SAE with smaller microspheres (<150–200 *μ*m). On the other hand, taking into account the size of the tumor feeders of our case, we thought that microspheres of larger diameters (≥500 *μ*m) would probably cause a very early and very proximal occlusion, with inadequate filling of the tumor's vascular bead and with increased risk of backflow.

We decided to combine our endovascular treatment with RFA, because our experience, as well as published data [3, 4], suggests that SAE alone may not always cause significant tumor shrinkage. This is particularly true for larger and fat-rich (at least 50% fat content) AMLs [4]. We also speculated that the ischemic effect of SAE might increase the safety of the subsequent ablative procedure, by limiting the risk for iatrogenic hemorrhage. It is also known that RFA is more effective when applied on devascularized tissue; otherwise, tissue cooling (caused by circulating blood) limits the heating effect of the ablation. Moreover, with RFA, we were able to target the upper and medial part of the tumor, which primarily caused compression of the IVC. The result of our combined treatment was a small reduction in the maximum tumor diameter, but a significant reduction in tumor volume and in the volume of the soft-tissue component. There was also a clinical benefit, since the patient symptoms improved and the reduction of tumor bulk was followed by resolution of the IVC thrombus. We acknowledge that our hypothesis regarding the relationship between the IVC thrombus and the mass effect of the AML is based only on imaging, and not on histopathology. Nevertheless, there was no other underlying cause of IVC thrombosis, and its resolution occurred only after tumor debulking. Contrary to our experience, almost all of the published cases of AML-related thrombus are caused by direct extension of aggressive AMLs (of epithelioid histology) into the renal vein and/or IVC [5]. Effective treatment of such cases is based on surgical (nephron-sparing surgery or nephrectomy plus thrombectomy) and not on interventional radiologic procedures.

RFA has emerged as a valuable treatment option of renal AML. RFA can be considered a nephron-sparing technique, since it can target the solid and vascular elements of the tumor, without damaging any normal renal tissue. The efficacy of RFA against AML has been assessed in relatively small series and mainly for small AMLs [6–8]: Castle et al. treated successfully 15 small renal AMLs with a low complication rate (13.3%) and complete disappearance of tumoral enhancement on CT, at a mean follow-up of 21 months [6]. However, changes in tumor size were not reported. Prevoo et al. reported a decrease in tumor size from 4.5 cm to 2.9 cm at 12 months after RFA of a sporadic AML in a patient with a solitary kidney. No complications occurred and no AML recurrence was observed during the 12-month follow-up [7]. Gregory et al. treated four large AMLs (maximal axis 6.1–32.4 cm). They reported no complications, no hemorrhagic events, and significant decrease in mean soft-tissue-to-total tumor ratio during a follow-up of 48 months. Nevertheless, the total tumor volume did not change significantly [8]. The same authors experienced problems of impedance when applying RF energy to the lipomatous parts of tumors and attributed the high impedance to the natural insulating properties of fat. We did not encounter similar problems, perhaps because we utilized different equipment and different ablation protocol and we primarily targeted the nonlipomatous parts of the tumor. Contrary to other authors, we used ultrasound instead of CT for guidance and monitoring of RFA. Since the CT-scanner in our institution is busy with diagnostic imaging, we usually reserve CT for tumors which cannot be safely and easily targeted by ultrasound. Moreover, insertion and manipulation of RF probes are often troublesome when the patient is in the CT-gantry. We recognize that sonographic monitoring of ablation is impaired by the echogenic “cloud” that appears around the RF electrode. Accurate depiction of untreated areas during ablation is crucial in the case of renal carcinoma (which should be, ideally, completely ablated); however the clinical context of our case was different and a detailed delineation of residual tumor was unnecessary. We believe that careful planning of the electrode trajectory, successful initial targeting of the lesion, and correct interpretation of intraprocedural sonographic findings can reduce the impact of the limitations of sonography.

Although experience regarding the combination of SAE and RFA is limited to a few cases, it appears that this combination is safe, feasible, and efficient. RFA may be applied after clinical failure of SAE, which may manifest as inadequate tumor shrinkage, tumor regrowth, or persistence of symptoms after SAE. Sooriakumaran et al. ablated 3 large sporadic AMLs (9–19 cm) previously treated with SAE and found a reduction in tumor mass of 20% and significantly reduced enhancement of the treated areas on CT or MRI after a median follow-up of 7.5 months [9]. Two of the patients of the aforementioned study of Gregory et al. also had a history of unsuccessful SAE [8].

Another remarkable feature of the presented case is that the multiple (and initially bilateral) AMLs of our patient were not associated with a congenital disorder, as shown by the clinical and imaging evaluation. No genetic testing was available. Multiple and bilateral AMLs may also be encountered (albeit rarely) on a sporadic basis [10].

We conclude that the combination of SAE and RFA may be a safe and effective option for the treatment of challenging cases of renal AMLs. This combination is worth being assessed in the context of a large study with adequate follow-up.
